# Improvements of Arboblend V2 Nature Characteristics through Depositing Thin Ceramic Layers

**DOI:** 10.3390/polym13213765

**Published:** 2021-10-30

**Authors:** Simona-Nicoleta Mazurchevici, Alina Marguta, Bogdan Istrate, Marcelin Benchea, Mihai Boca, Dumitru Nedelcu

**Affiliations:** 1 Department of Machine, Manufacturing Technology, ”Gheorghe Asachi” Technical University of Iasi, 700050 Iasi, Romania; simona0nikoleta@gmail.com (S.-N.M.); alinamarguta@yahoo.com (A.M.); mihaitzaboca@yahoo.com (M.B.); 2 Department of Mechanical Engineering, Mechatronics and Robotics, Faculty of Mechanical Engineering, “Gheorghe Asachi” Technical University of Iasi, 700050 Iasi, Romania; bogdan.istrate@tuiasi.ro (B.I.); marcelin.benchea@tuiasi.ro (M.B.); 3 Mechanical Engineering Department, Technical Sciences Academy of Romania, 030167 Bucharest, Romania

**Keywords:** biodegradable thermoplastic, coating, Amdry 6420, Metco 143, Metco 136F, hardness, adhesion, structure

## Abstract

The paper aims to investigate the behavior of Arboblend V2 Nature biopolymer samples covered with three ceramic powders, Amdry 6420 (Cr_2_O_3_), Metco 143 (ZrO_2_ 18TiO_2_ 10Y_2_O_3_) and Metco 136F (Cr_2_O_3_-xSiO_2_-yTiO_2_). The coated samples were obtained by injection molding, and the micropowder deposition was achieved by using the Atmospheric Plasma Spray (APS) method, with varied thickness layers. The present study will only describe the results for nine-layer deposition because, as the number of layers’ increases, the surface quality and mechanical/thermal characteristics such as wear, hardness and thermal resistance are also increased. The followed determinations were conducted: the adhesion strength, hardness on a microscopic scale by micro-indentation, thermal analysis and structural and morphological analysis. The structural analysis has highlighted a uniform deposition for the ZrO_2_ 18TiO_2_ 10Y_2_O_3_ layer, but for the layers that contained Cr_2_O_3_ ceramic microparticles, the deposition was not completely uniform. The thermal analysis revealed structural stability up to a temperature of 230 °C, the major degradation of the biopolymer matrix taking place at a temperature around 344 °C. The samples’ crystalline structure as well as the presence of the Cr_2_O_3_ compound significantly influenced the micro-indentation and scratch analysis responses. The novelty of this study is given by itself the coating of the Arboblend V2 Nature biopolymer (as base material), with ceramic microparticles as the micropowder coating material. Following the undertaken study, the increase in the mechanical, tribological and thermal characteristics of the samples recommend all three coated biopolymer samples as suitable for operating in harsh conditions, such as the automotive industry, in order to replace plastic materials.

## 1. Introduction

The thermal coating process finds its applicability in areas such as the automotive, aerospace and naval industries in order to improve the corrosion resistance, wear resistance and lifetime of the equipment part as follows: thermal barrier coatings for components that operate in severe thermal conditions (turbine blades, fuel parts, vanes) with the role of increasing their life by improving resistance to oxidation, heat and corrosion [1]; hard metal coatings such as tungsten and chromium carbide are commonly used to increase the wear and corrosion resistance of the parts surfaces [2]; corrosion resistance after heat treatment of a Cr_3_C_2_–NiCr coating showed significant improvement due to both the microstructural changes and the presence of a metallurgical bond at the coating–substrate interface [3]; titanium plasma spraying (TPS) used on polymers as polyethylene/polyamide highlighted good mechanical properties and increased hardness [4].

One of the most common thermal deposition techniques is Atmospheric Plasma Spray (APS), and the coatings obtained by zirconium and chromium ceramic layer depositions are widely spread materials in mechanical applications [5]. The main drawbacks of these coatings are given by the micro-cracks, isolation inhomogeneity and residual stresses (appears during cooling process) [6].

The Al_2_O_3_ (aluminium oxide (alumina)) powder with high purity used for coating, usually applied by the plasma spray method, presents electrical insulation in terms of thermal conductivity and dielectric characteristics. Furthermore, the coatings are wear-resistant, have higher hardness, higher temperature stability and are chemically inert. The ceramic coating is suitable for electrical, electronic and semiconductor manufacturing tool applications, electrostatic chucks and capacitors, vacuum chamber lines, etc. The high purity of the material ensures that it will not contaminate the semi-conductor components [7]. Nickel, zirconium, aluminium, molybdenum and chromium, as powder elements, are a good choice for applications that require high toughness with moderate resistance to fretting, erosion and scuffing. Zinc-based coatings are widespread in applications that require increased corrosion resistance, but if this chemical element is not alloyed with other elements such as tungsten carbide or nickel, it has low mechanical characteristics [8]. The degradation of the Zn–polymer interface has been studied by the authors of the paper [9], and they point out that the delamination can be inhibited by CO_2_ gas in a humid environment. Inhibition depends very much on the polymer matrix’s affinity for carbon dioxide. Other researchers such as [10] have used nano-zinc-oxide (5 wt %) and epoxy acrylate in order to obtain a corrosion protection coating for mild steel panels.

It is well known that the addition of ceramic particles, metals particles and biopolymers as a substrate provides a combination of properties of all three types of materials: biopolymer matrices and metallic and ceramic reinforcement components. This may result in the improvement of the physical and mechanical properties of the composite [11].

In the literature, the applications of function coatings refer mainly to corrosion protection characteristic, and there is a limited emphasis on equally important properties—for example, mechanical robustness, which is significant for determining the applicability of developed coatings. However, there are discussions on the principal working theories, procedures for preparation, performance investigations and applications of superhydrophobic coatings, for instance: a viable preventative method for controlling metal corrosion due to their mechanical stability and durability, and the shortcoming consists in the ability of maintaining this characteristic for a prolonged period of time [12]; corrosion inhibition of metallic materials with the help of smart coatings involves difficulties in achieving some features as thermal stability, resistances to scratching and strong chemical acids, high optical transmission, in situ healing, etc. [13]; coating of stainless steel and titanium bipolar plates in order to improve the corrosion resistance and electrical conductivity in PEMFC (proton-exchange membrane fuel cells), which has, for the moment as the main impediment in the large-scale spread of the product, a high manufacturing cost [14]; excellent anticorrosion durability obtained by coating the polyaniline–graphene oxide composite with zinc-based waterborne [15]; anticorrosive coatings in the marine field face the degradation, loss of adhesion and failure of coating systems [16]; metallic substrates (aluminum alloy) coated with active corrosion protection systems (with self-healing ability) such as silica−zirconia nanoparticles highlighted long-term corrosion protection and the ability to self-heal defects, and these characteristics are obtained by rigorous control of the inhibitor regular release at the moment when the corrosion process begins to arise [17]. A comparative review that accounts for all factors, including the durability and other mechanical properties, is essential for understanding the applicability of an advanced coating at a practical level.

In addition, another area targeted by coating researchers is related to composites based on polymer matrices with the inclusion of magnetic nano-sized particles: Polydimethylsiloxane (PDMS) coated with different concentrations of nanosized Ni@C core-shell [18]; Ni-silicone elastomagnetic composites [19]; polyacrylamide-based hydrogels coated with Ni ferrite [20]; polyetherurethane (TFX) and a biodegradable multiblock copolymer (PDC) with poly(p-dioxanone) as hard segment and poly(ε-caprolactone) as soft segment were investigated as matrix component, coated with iron oxide particles [21]; and oligo(ε-caprolactone)dimethacrylate/butyl acrylate, coated with Fe_3_O_4_ [22]; carbon-fiber–epoxy composites coated with two thermal ceramic particles (lass flakes and aluminum titanate) in order to create a thermal barrier for the substrate [23]. The interest of researchers was focused mainly on elastomagnetic effects [19] and the wide prospects of applications as follows: Ni ferrite with a highly organized structure as humidity sensors [20]; magnetic nanoparticles for practical applications which involve sensors and biosensors [24]; magnetoresistive sensors for applications where the ultimate field detection limits are required or as readers in hard disk drives [25]; Mg substitution on Ni-ferrite ceramics with applications in biomedicine, gas detection, heterogeneous catalysis, adsorption, etc. [26]; shape-memory materials through the inductive heating of magnetic nanoparticles in thermoplastic polymers [21]; and the incorporation of surface-modified superparamagnetic nanoparticles into a polymer matrix [22]. These materials also demonstrate prospects for biomedicine: drug delivery, hyperthermia, magnetic resonance imaging contrast enhancement [27] and the manipulation of cell membranes [28]; recording media and high-frequency applications—electromagnetic-wave-absorption materials [29], microwave absorption [30] and gigahertz microwave absorption [31].

In this paper, the authors have analysed three types of plasma jet coatings for intermediate layers (micrometallic powder) and one type of ceramic coating (aluminium oxide). The coatings were made on Arboblend V2 Nature substrate materials. The samples were obtained using injection moulding, and the method used for coating was atmospheric plasma spraying.

The registered trademark Arboblend^®^, developed by scientists and engineers from the German company Tecnaro in collaboration with those from the Fraunhofer Institute for Chemical Technology, is a 100% biodegradable biopolymer [32], and a part of the current research group has investigated the behavior of samples covered with metallic intermediate layer and ceramic final layer in the past but not at such a deep level and not following an experimental plan [33].

The aim of the manuscript was to obtain a new material with improved properties that can then be used successfully as a substitute for synthetic plastics in the automotive industry. Given this objective, the biodegradable material Arboblend V2 Naure was chosen, for which the research team previously studied the properties. The next step was to realize the coating with ceramic microparticles by using the APS method, after which the same characteristics were studied. The present study is not found in the research activity of other authors, so the proposed research through both technology and experimental results is constituted as a novelty element.

The introduction section provides a coatings overview. This chapter is followed by a description of the materials and methods used in the analysis of the coated samples. The results of the experimental research are presented and commented on in Section 3.4, and the conclusions part suggestively presents general comments on the main obtained results.

## 2. Materials and Methods

The thermoplastic material selected to be coated with ceramic microparticles was Arboblend V2 Nature. According to the information provided by the producers but also to some studies from the specialized literature [32,34,35], the basic matrix of the polymer is lignin, this being extracted from annual vegetable plants, so it is not necessary to use wood raw materials that require dozens of years or even longer to reach maturity and be used in the forestry and paper industry. This is important to mention because another source of lignin used for this material comes from its extraction from the paper industry waste. In addition, the Arboblend V2 Nature structure can contain a significant amount of polylactic acid (PLA—also biodegradable polyester) and other constituents such as bio-polyamides (bio-PA), cellulose, natural vegetable fibers and, for processing in good conditions, contains a small amount of natural additives (resins, waxes, shellac) [33,36,37].

Obtaining the necessary samples for coating with ceramic layers was realized by injection in the mold using the SZ-600H equipment (SHEN ZHOU, Zhangjiagang, China). The dimensions of the samples were (70 × 50 × 10) mm^3^. The following technological parameters were used for injection: material melting temperature—165 °C; injection pressure—100 MPa; injection speed—80 m/min; cooling time—30 s.

The preparation of the rectangular biopolymer samples consisted in the fixed adhesion between metal strips, followed by the blasting and removal of impurities in order to obtain a surface roughness that was as low as possible. The final stage of preparation was degreasing with ethyl alcohol.

Atmospheric plasma spray (APS) technology (SPRAYWIZARD-9MCE, Sultzer-Metco, Westbury, New Yorkxz, USA/9MBspraying gun) was used to cover the injected samples. The technological coverage parameters used are shown in Table 1.

The deposition rate of the microparticles was constant. The thickness of the deposited ceramic layer was of the micrometers order and for the thermal control of the samples’ melting temperature, a laser pyrometer was used throughout the process.

Three ceramic powders were used for the coating: Amdry 6420 (Cr_2_O_3_), Metco 143 (Cr_2_O_3_-xSiO_2_-yTiO_2_) and Metco 136F (ZrO_2_ 18TiO_2_ 10Y_2_O_3_). The three micropowders were deposited on three samples injected from Arboblend V2 Nature. On each sample, a distinct number of passes was made, namely, 5, 7 and 9 passes, in order to study the improvement or not of the mechanical characteristics with the increase in the deposited ceramic layer. However, this manuscript will only present the results for the samples obtained by performing 9 passes, because the objective of the paper is to highlight the uniformity and homogeneity of the deposited layers, which is not entirely revealed by the samples obtained with 3 and 5 passes. The experimental plan used to cover the samples with ceramic micropowders is highlighted in Table 2.

The ceramic micro powders were purchased from the Oerlikon Metco manufacturer (Bella Vista, New South Wales, Australia). The microparticles dimensions and shapes are varied as follows [38]:

- Chromium Oxide Thermal Spray Powder, Amdry 6420 (Cr_2_O_3_): angular, blocky morphology, (10–105) µm;
- Chromia–Silica composite powder, Metco 136F (Cr_2_O_3_-xSiO_2_-yTiO_2_): irregular or angular/blocky morphology, (9–110) µm, Cr_2_O_3_—Balance; SiO_2_—(3.0–4.5)%; TiO_2_ < 4.0%;
- Zirconia–Titania–Yttria Composite Powder, Metco™ 143 (ZrO_2_ 18TiO_2_ 10Y_2_O_3_): spheroidal morphology; typically particle size between (3–40) μm;
- The microindentation and scratch tests used the CETR UMT-2 microtribometer (Universal Materials Tester, CETR^®^, Campbell, SUA). Test conditions were as follows:
- Scratch analysis: a blade with a tip of 0.4 mm (radius at the tip) was used, the samples were fixed on the table and during the test samples were pressed with a vertical force of 10 N NVIDIA blade, moving the table over a distance of 10 mm in 60 s, and the test speed was 0.167 mm/s. The software performed the automatic test and recorded the following parameters: vertical force F_z_, horizontal force F_x_, time and distance of movement in the horizontal direction Y (of the fixing mass of the sample).
- Microindentation test: the Rockwell type indenter was used (cone with diamond tip having an angle of 120° and a radius at the peak of 200 microns), the samples were fixed on the table and during the test samples were pressed with a vertical force of 10 N (with steps/times described in work). Three samples of each type of powder were tested in order to be able to calculate the parameters statistically with the highest possible accuracy (hardness and Young’s modulus). The software performed the automatic test and recorded the following parameters: vertical force F_z_, time and vertical travel distance C of the indenter (with capacitive sensor). Other process parameters were loading time—30 s; holding time—15 s; unloading time—30 s; and sensor of (0.2–20) N. Microindentation tests involved testing three samples for each type of ceramic powder in order to confirm experimental repeatability. The average values were obtained by calculating the arithmetic average, and the standard deviation highlights the variation in a set of numbers compared to the calculated average value.

In order to determine the thermal, structural and morphological behavior, only the samples with 9 successive passes were selected for this study: sample 3 coated with Zirconia–Titania–Yttria Composite Powder, Metco™ 143, further noted with P3–143–9 passes; sample 6 coated with Chromium Oxide powder, Amdry 6420, noted with P6–6420–9 passes; sample 9 coated with Chromia–Silica composite powder, Metco 136F, noted with P9–136–9 passes. The used equipments for these analyzes were as follows:

Differential scanning calorimetry *(DSC)* was performed on a DSC 200 F3 Maia differential scanning calorimeter (NETZSCH-Gerätebau GmbH, Selb, Germany). The calibration of the device was realised in accordance with mercury (Hg), zinc (Zn), Indium (In), Tin (Sn) and Bismuth (Bi) standards. The mass of the analyzed samples was less than 30 mg. The experiments were analyzed in the atmosphere of inert gas (Ar). In this experiment, a sample and a reference (an empty crucible) were subjected to the same temperature program. The temperature program consisted of heating from room temperature (RT ≈ 20 °C) to 200 °C, then cooling from this temperature to RT. The heating and cooling speed used was 10 K/min. During the experiment, the reference and sample temperatures were measured and the temperature difference recorded between the two was converted into heat flux. The recorded thermograms were then evaluated using Proteus software (provided by NETZSCH). The tangent method was used to determine the transition temperatures. The area was determined by using a rectilinear baseline. Temperature of the transformation beginning (T_onset_), temperature assigned to the peak (T_peak_), temperature at the end of the transformation (T_end_) and the amount of absorbed or dissipated heat were determined.

Thermogravimetric curves (*TG*), derived thermogravimetric curves (*DTG*) and differential thermal analyzes (*DTA*), were determined using Mettler Toledo TGA/SDTA 851 equipment. The mass of samples subjected to thermal decomposition was between 2.9 and 3.9 mg. It was worked in an air atmosphere with a flow rate of 20 cm^3^/min. The study was realized in the temperature range of 25–700 °C using a heating rate of 10 °C/min. The processing of thermogravimetric curves was performed with the STARe SW 9.10 software from Mettler Toledo (Columbus, OH, USA). As in the case of DSC analysis, the beginning (T_onset_), maximum (T_peak_) and end (T_end_) temperatures of each thermal degradation stage were determined. For each identified stage, it is indicated the loss mass percentage residue (W%).

SEM structural analysis (Scanning Electron Microscopy) was performed on the QUANTA 200 3D electron microscope (FEI Company, Fremont, CA, USA). Micrographic maps of the samples coated with ceramic micropowders were made on their surface to observe mainly the uniformity of the deposition. The main parameters considered were the following: the pressure inside the microscope chamber—60 Pa; detector—(Large Field Detector) for the analysis of non-conductive samples such as polymers, textile fibers, powders, etc.; tilt angle—0°; secondary electron acceleration voltage—20 Kv; working distance—15 mm; magnification power—500×–2000×.

To determine the chemical elements that appeared with the deposition of the ceramic layers, an Energy-dispersive X-ray spectroscopy (EDX) together with a SEM was performed. The SEM equipment was VegaTescan LMHII (TESCAN ORSAY HOLDING, Kohoutovice, Czech Republic) with EDX detector X Flash 6I10 from Bruker, Germany, using Esprit 2.2 software. The type of EDX analysis was in-line in order to capture as accurately as possible the difference in chemical composition between the resulted microceramic layer and substrate material.

X-ray diffraction analysis (XRD) was performed with the X’Pert Pro MRD X-ray diffractometer, which has a Cu kα anode X-ray tube, λ–1.54 Å, Panalytical equipment (PANalytical, Almelo, the Netherlands), on which a voltage of 45 kV was applied, the variation of the diffraction angle (2θ) being between 10 and 90°. Two X’Pert Data Collector programs were used to process the data and make the diagrams, namely X’Pert High Score Plus version number 3 and X’Pert Data Viewer version number 2.2 g (Malvern Panalytical, Malvern, UK). This analysis aimed to identify the existence of crystallization phases specific to ceramic micropowders deposited on the surface of the samples from Arboblend V2 Nature. The identification of the crystallization phases was performed by comparing the obtained data with those from the scientific literature.

The chemical composition analyses were performed in five distinct points, and an overall average composition was made with the help of Minitab software.

## 3. Results and Discussion

### 3.1. DSC Analyse

In order to establish the physical transformations that take place during the gradual heating of the Arboblend V2 Nature samples coated with ceramic micropowders, a DSC analysis was performed. Three distinct samples were used, one from each type of powder: P3–143–9 passes; P6–6420–9 passes; and P9–136–9 passes. The sample size was less than 5 mm; their mass was less than 20 mg.

During the heating of the three selected samples, three transformations were highlighted, two of them endothermic (I^st^ and III^rd^) and one exothermic (II^nd^), the same thermal behavior being highlighted for the sample injected from Arboblend V2 Nature but not covered with a ceramic layer [34,35].

**Figure 1:**
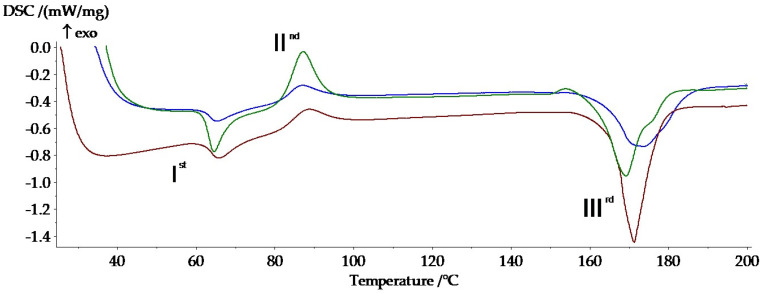
Highlighting the thermal behaviors of the tested samples: I^st^—first transformation; II^nd^—second transformation; III^rd^—third transformation; P3–143–9 passes (green), P6–6420–9 passes (blue), P9–136–9 passes (red).

The variation in the heat flow in relation to the recorded temperature for the three phase transformations of each analyzed sample is shown in Figure 1. In order to highlight the possible different thermal compartments of the coated samples, the three signals were overlapped.

Both Figure 1 and Table 3 reveal changes in the temperatures at which the phase transformations take place. This aspect can be attributed to the small but still existing mass difference between the three analyzed samples (P3–143–9 passes 12.6 mg, P6–6420–9 passes 16.7 mg, P9–136–9 14.5 mg). At the same time, the amount of heat absorbed or dissipated is slightly different [39]. Another reason that could have generated this difference is the thickness of the deposited layer with the completion of the nine passes. As the dimensions of the microparticles differ in the case of the three ceramic powders, it is expected that the powder, whose microparticles are larger, will form a thicker layer, and thus the phase transitions will take place at slightly higher temperatures and the amount of absorbed or dissipated heat will be lower.

The critical temperatures of the three transformations are as follows: T_onset_ is the starting temperature; T_peak_ is the middle temperature; T_end_ is the finishing temperature (determined using the tangent method); and ΔH/m is the amount of dissipated/absorbed heat (using a rectilinear baseline).

Analyzing the first phase transformation, it is observed that the three samples register an endothermic maximum around 65 °C, a slightly lower transition temperature (64.7 °C) that the P3–143–9 passes sample (Zirconia–Titania–Yttria Composite Powder), whose powder has a smaller granulation. The first peak can be associated with a slow monotropic transformation of the solid-solid type and of some metastable crystals [40], which takes place with reduced heat absorption, −8.81 kJ/kg for P3–143–9 passes, −4.29 kJ/kg for the P6–6420–9 passes and −5.57 kJ/kg in the case of the P9–136–9 passes. The variation in the absorbed heat can be attributed, first of all, to the thickness of the deposited layers but also to the mass difference of the analyzed samples.

The second peak takes place around the temperature of 86 °C, the powder with the highest granulation, the P9–136–9 passes, registering an increase in the transformation with 1.5 °C higher than the other two covered samples. The exothermic peak can be associated with the base biopolymer crystallization or with the reticular reorganization of lignin, the basic matrix of the biopolymer [39].

The third peak occurs with considerable heat absorption, in the case of all analyzed samples: −40.1 kJ/kg for the sample with smaller microparticles (P3–143–9 passes) and −48.38 kJ/kg for the sample with higher granulation (P9–136–9 passes). The endothermic transformation is attributed to the melting of the Arboblend V2 Nature biopolymer around the temperature of 170 °C, with small variations depending on the size of the ceramic microparticles.

### 3.2. TG Analyses

**Figure 2:**
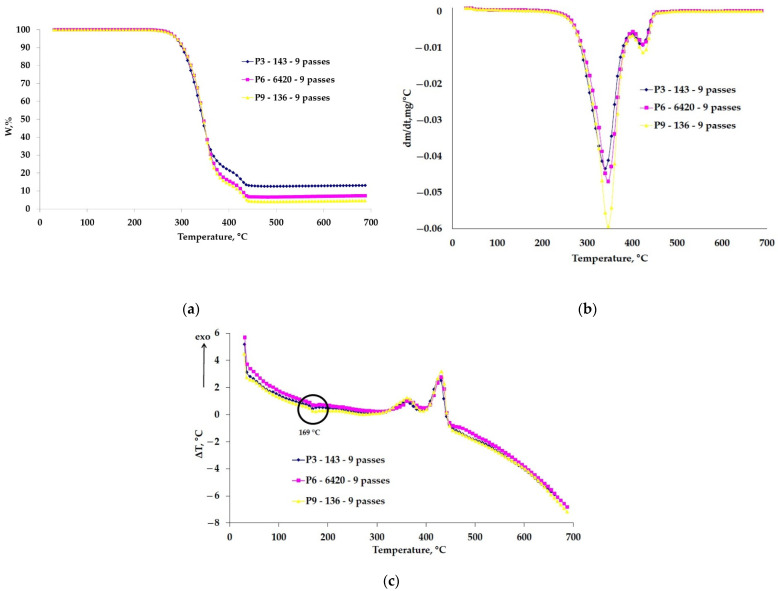
Thermogravimetric curves of the coated samples: (**a**) TG, (**b**) DTG and (**c**) DTA.

Knowing the thermal stability of Arboblend V2 Nature samples coated with ceramic powders is essential because their use in applications that require operation in severe working conditions, whether it is wear resistance or thermal resistance, requires the study of thermogravimetric behavior. It is desirable that the coating with ceramic microparticles increases the mechanical characteristics but also the thermal stability. Figure 2 compares the thermogravimetric (TG), derived thermogravimetric (DTG) and differential thermal (DTA) curves for the three samples coated with ceramic layers made from nine successive passes.

The main thermogravimetric characteristics of the P3–143–9 passes, P6–6420–9 passes and P9–136–9 passes samples are presented in Table 4.

The curves obtained from the TG analysis were overlapped to highlight their difference in behavior, and the thermal stability of the coated samples is similar (Figure 2).

The three coated samples with different ceramic layers highlight two decomposition stages, the first recorded being around a temperature of 345 °C, with a significant mass loss of over 85%: this decomposition is attributed to the structural degradation of the basic constituent of the material, lignin. This stage consists of the formation of aromatic hydrocarbons, guaiacyl-/syringyl-type and hydroxy-phenolic compounds and more [40]. According to the manufacturer [32], another constituent contained by the analyzed biopolymer is PLA, which decomposes in considerable proportions in this temperature range [41,42]. According to the literature [43,44], PLA and pure lignin degrade completely up to a temperature of 500 °C.

In the second stage with a T_peak_ around 425 °C, there is a mass loss in a percentage much lower than 10%, attributed to the thermal oxidation of the carbonic residue that appeared from the pyrolysis of lignin and/or PLA but also of another biodegradable constituent of the biopolymer that was introduced by the manufacturer as a binder (resin, wax, shellac, etc.) [33]. At a temperature of 700 °C, depending on the used ceramic powder type, a certain amount of residual mass is found. It is observed that the sample P6–6420–9 passes has the highest percentage of residue, 6.6%, most likely due to the higher amount of microparticles than, for example, in the case of the P3–143–9 passes sample, where the amount of ceramic powder deposited is much lower. The ceramic powders at the end of the analysis temperature have not yet reached the melting point of approximately 2500 ° C, their working temperature varying from 540 °C (P6–6420–9, P9–136–9 passes) to 980 °C (P3–143–9 passes) [38]. In addition, inorganic substances that are found in the composition of the biopolymer are very likely to be part of the residual mass [45].

Figure 2c shows the DTA curves where the melting temperature of Arboblend V2 Nature can be observed, 169 °C, very close to the values obtained by calorimetric analysis, especially in the case of the P3–143–9 passes sample.

### 3.3. Surface and Structure Analysis of Coated Samples

#### 3.3.1. SEM Analysis

**Figure 3:**
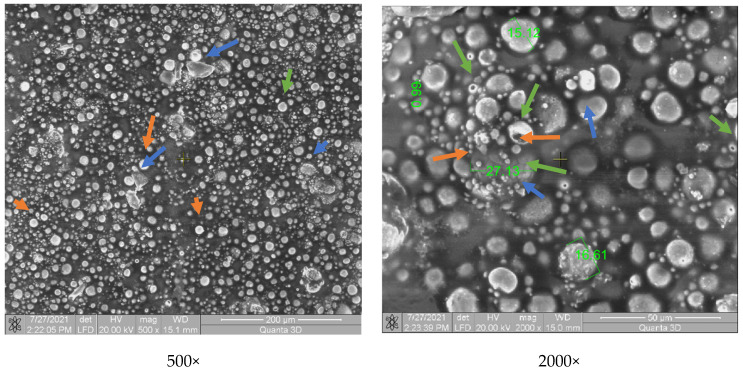
SEM analysis of the P3–143–9 passes samples: Zirconium dioxide (bearing balls)—orange arrow; Titanium dioxide—blue arrow; Yttrium oxide—green arrow.

Figure 3 shows the morphological aspect of the Arboblend V2 Nature material coated with Metco ™ 143 (ZrO_2_ 18TiO_2_ 10Y_2_O_3_). A uniform coating of the biopolymer mass is observed. The coating consists of spherical component particles having a dimensions variation between 1 and 27 µm. The particles retain their spherical shape due to the very rapid cooling on contact with the base matrix. They do not flatten in the form of splats as is conventional in the case of coatings on metal substrate [46]. The fact that the basic matrix contains various particles in shape and size, in large quantities and evenly distributed, leads to an increase in mechanical properties.

Yttrium oxide (green arrow, Figure 3) shows a porous spherical morphology in the form of a sintered agglomerate.

**Figure 4:**
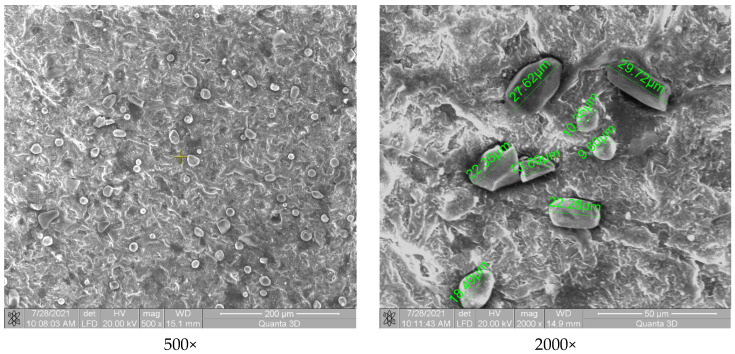
SEM analysis of the P6–6420–9 passes samples.

Figure 4 shows the polymer matrix containing particles from the coating formed by chromium oxide. Some of these particles are heterogeneously distributed, and another part is embedded in the polymeric structure. Their size varies from 18 µm to 30 µm, and they have rectangular shapes specific to chromium oxide. The spherical microparticles can be attributed to the presence of Fe_2_O_3_ and SiO_2_, which are released in small quantities in the structure of the Amdry 6420 powder, maximum 0.4% and 0.45%, respectively. Compared to the P3–143–9 passes sample, the material incorporated a smaller amount of powder appearance, which represents the lower capacity of the coating and embedding chromium oxide in the polymeric structure.

**Figure 5:**
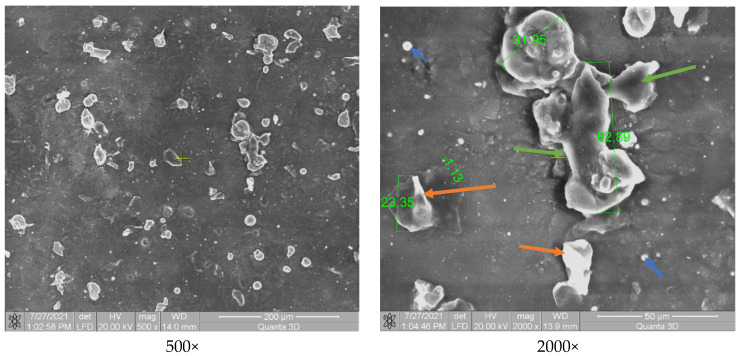
SEM analysis of the P9–136–9 passes samples.

The coating of chromium oxide, silicon oxide and titanium oxide (Cr_2_O_3_-xSiO_2_-yTiO_2_, Figure 5) highlights a relatively uneven distribution of microparticles, similar to the situation presented in Figure 4. The particles are of different shapes with mostly polyhedral appearance (TiO_2_—green arrow) but there are also spherical (SiO_2_—blue arrow) and rectangular (Cr_2_O_3_—orange arrow) structures, their dimensions varying between 1 and 63 µm. The particles are embedded in the polymer mass. Both Figure 4 and Figure 5 show chromium oxide, and this compound does not have a better adhesion compared to the sample coated with P3–143–9 passes (Figure 3), which does not contain chromium oxide.

#### 3.3.2. EDX Analysis

In order to observe the presence of the deposited ceramic layers, the line EDX analysis was performed together with the SEM analysis of the P3–143–9 passes, P6–6420–9 passes and P9–136–9 passes samples. This analysis series had as the point of study the edge area of the samples in order to highlight as accurately as possible the presence, distribution and concentration of the chemical elements which form the ceramic microlayers and also the biopolymeric support.

**Figure 6:**
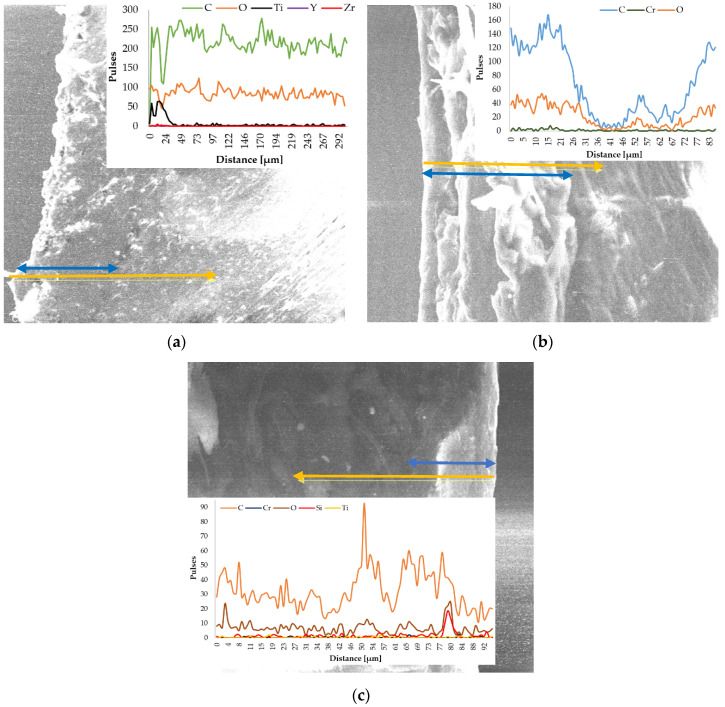
In line EDX analysis of the coated samples. (**a**) P3–143–9 passes, (**b**) P6–6420–9 passes, (**c**) P9–136–9 passes.

The EDX in-line analysis (Figure 6—yellow arrow) of all samples reflects the abundant presence of two chemical elements, carbon and oxygen. Their existence is closely related to the chemical composition of the biopolymer matrix which presents, according to the previous determinations, C and O in similar mass proportions, 48 ± 0.02% and 52 ± 0.02%, respectively [35]. The acquisition of data is a difficult one because the polymer matrix is not an electrical conductor, so the electrons coming out of the material are of much lower intensity than, for example, in the case of an analyzed metallic material.

For the sample covered with Zirconia–Titania–Yttria Composite Powder (Figure 6a), the presence of ceramic microparticles in the first 20 µm is observed, an area that we consider to coincide with the thickness of the deposited ±0.02 ceramic layer (blue arrow). Titanium is the chemical element found in the most significant amount in the area of the ceramic layer, but the other two elements are highlighted by the graphic. The presence of a small amount of Zr and Ti during the entire test distance is due to the microparticles that came off of the brittle ceramic layer in the moment of EDX preparation. The higher amount of carbon than oxygen is attributed to the carbides that form during the embedding of molten microparticles in the solid (cold) polymeric matrix, carbides that provide surface hardness. Oxides are also formed but in a much smaller amount.

Chromium being a hard metallic element during the preparation of the P6–6420–9 passes sample, this one is visible over the entire analyzed surface; however, it can be observed that in the first part of the analyzed distance (first 20 µm), the amount of chromium is higher (Figure 6b). As in the case of the P3–143–9 passes sample, the amount of carbon is higher than that of oxygen. In the 35–75 µm interval, the graph highlights a steep decrease in the amount of elements reflected by the sample, but most likely this can be attributed to the transition between the deposited layer and the biopolymer support.

For the P9–136–9 passes sample (Figure 6c), the in–line analysis reveals the presence of the ceramic layer on the right side of the SEM image (last 15 µm), and the graph reflects the existence of the micropowder chemical elements. Silicon being a semiconductor chemical element reflects its presence in the structure of the ceramic layer much more than the than the other constituents. The Cr, Ti and O of the composite micropowders were highlighted by the analyzed sample.

#### 3.3.3. XRD Analysis

The main purpose of the XRD analysis was to determine the structure of the samples made of Arboblend V2 Nature and coated with ceramic micropowders, Amdry 6420 (Cr_2_O_3_), Metco 143 (ZrO_2_ 18TiO_2_ 10Y_2_O_3_) and Metco 136F (Cr_2_O_3_-xSiO_2_-yTiO_2_), but also to identify possible crystal phases.

**Figure 7:**
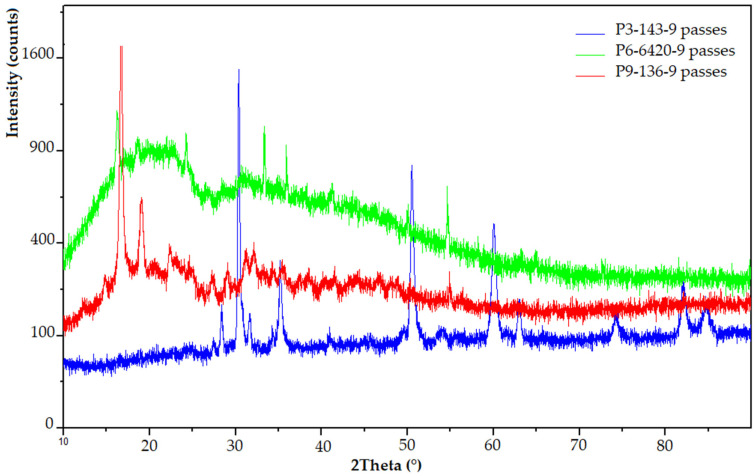
XRD analysis for ceramic coated samples: P3–143–9 passes (blue), P6–6420–9 passes (green), P9–136–9 passes (red).

Figure 7 shows the phase diffractograms for the three samples with distinct ceramic coatings: P3–143–9 passes, P6–6420–9 passes and P9–136–9 passes. It can be seen that two of the three samples have a crystalline structure (P3–143–9 passes, P9–136–9 passes) highlighted by specific peaks, and the third (P6–6420–9 passes green diffractogram) has a semi- crystalline structure with the presence of small peaks of chromium oxide at four distinct 2θ angles, 24.25°, 33.39°, 35.88° and 54.61°, respectively, of low intensity, 1036 to 1356 [47,48,49]. The major peak registered at 16.73°, with a diffraction intensity of 2643, which, according to the literature, may be associated with the presence of polylactic acid in its the chemical composition ((C_3_H_4_O_2_)n) [50,51]. The presence of this compound is not accidental because it is due to the fact that the thickness of the deposited layer is very thin (small microparticles), thus, the equipment is detecting one of the basic material constituents.

In the case of the other two coatings, the presence of predominant peaks is observed, which correspond to the crystallization of certain compounds as follows:

- The P3–143–9 passes sample (blue diffractogram) has a strong crystalline aspect due to the large amount of zirconium dioxide particles present in the structure of the ceramic powder. Thus, to the ZrO_2_ compound can be assigned the peaks from 2θ = 31.14°, 38.43°, 60°, 82° and 84.78° [46,52,53]. Titanium dioxide, as in the case of the P9–136–9 passes sample, is found at angles of 27.43°, 28.34°, 63.02° and 74.41° [54,55]. However, the intensity of the diffraction peaks is quite low, the highest being registered in the case of the 2θ = 28.34° angle. The low angle from 2-theta = 43°, was identified as the specific angle of Y_2_O_3_ crystallization [56].
- The P9–136–9 passes sample (red diffractogram) shows diffraction maxima associated with the presence of the polymer matrix, which has in its structure polylactic acid (16.73°) and lignin or natural fibers (19.04°) [53,57]. Diffraction angles corresponding to the coating with ceramic micropowder are also visible: The specific peaks to Cr_2_O_3_ crystallization at 2-theta angles of low intensity are 30.35°, 31.70°, 35.16°, 50.48° and 54.12° [47,48,49,58]. For SiO_2_ microspheres, a peak located at about 2θ = 22.5 is observed [59]. No other diffraction peaks can be detected for this compound. According to the literature [54,55], the diffraction angles that can be attributed to the titanium dioxide (TiO_2_) present are at 27.33° and 32.13° in the case of Metco 136F micropowder.

### 3.4. Scratch Analysis

The scratch test was performed in order to evaluate the adhesion of the hard (ceramic) coatings made on the surface of the Arboblend V2 Nature biopolymeric material.

**Figure 8:**
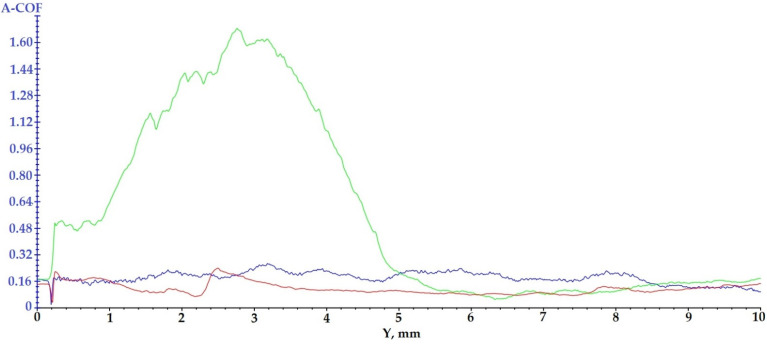
Results regarding the scratching behavior of the samples coated with ceramic layers: blue curve—P3–143–9 passes; green curve—P6–6420–9 passes; red curve—P9–136–9 passes.

Analyzing the curves presented in Figure 8, it is observed that one of them, the green curve (P6–6420–9 passes), shows a sudden and gradual transition of the apparent friction coefficient (A-COF), which means that the adhesion between the deposited thin layer and the polymeric material is better than in the case of the other two coatings due to the presence of chromium oxide. The other two tests have a good scratching behavior, but the P3–143–9 passes test (blue curve), recorded higher A-COF values than in the case of the P9–136–9 passes test.

The more peaks that appear in the variation of the apparent friction coefficient, the better the adhesion between the deposited layer and the polymeric material is.

For the P6–6420–9 passes sample, a high amplitude peak of A-COF is registered at the beginning of the test; the explanation for this would be related to the deposition granulation. It is very possible that the tip of the cutting tool (pin) has hung an area of deposited material with a larger granulation.

**Figure 9:**
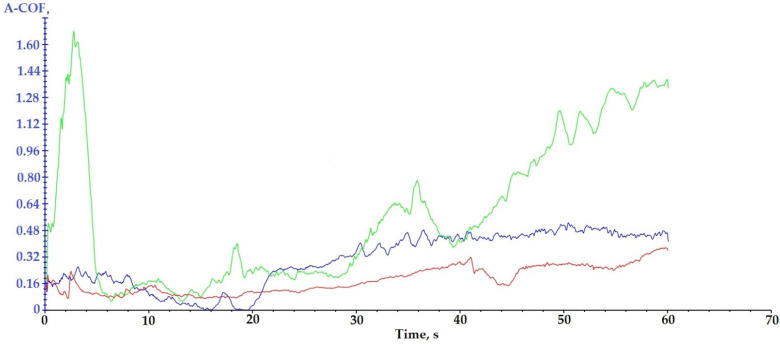
A-COF variation with test time for samples coated with ceramic micropowders: (blue) P3–143–9 passes; (green) P6–6420–9 passes; (red) P9–136–9 passes.

The samples injected from Arboblend V2 Nature and coated with ceramic micropowders showed the following behavior during the 60 s of testing (Figure 9): For sample P3–143–9 passes, blue curve, an increase in A-COF is observed in the first 4 s, after which its value begins to decrease sharply until the 16 s when it registers an increase followed quickly by a decrease. Starting with at 19 s, the average value of A-COF increases and begins to stabilize, reaching a maximum value of 0.53 at 50 s. The mean value of A-COF was 0.29 ± 0.16. The behavior of the sample coated with Zirconia–Titania–Yttria composite powder is a typical one, and the coatings in the first part of the test register variations of A-COF in order to later stabilize. Sample P6–6420–9 passes, green curve, reflects a completely different behavior from the first test. In the first part of scratching, the first 3 s, it reaches the maximum value of A-COF at 1.62, followed by a sudden decrease until 6 s. Next, the sample registers two fluctuations, and starting at 40 s, the A-COF value begins to increase, at the end of the 60 s reaching the value of 1.37. These fluctuations can be attributed to the variable dimensions (9–30 µm) of the microparticles that constitute the ceramic powder. The progressive increase recorded in the last 20 s of testing reflects the fact that the test pin detached ceramic microparticles from the sample surface, thus gradually becoming more and more rough. The mean value of A-COF for this sample, 0.56 ± 0.42, is the highest compared to the other two tested samples. The last test subjected to tribological determination, P9–136–9 passes, red curve, as well as the previous test, records fluctuations throughout the test with the A-COF value at the end of the test reaching a maximum of 0.37. The average value of A-COF for this sample is the lowest, 0.18 ± 0.08. The value registered is very close to that of the injected samples and not covered with ceramic layer, 0.16 for rotational determinations and 0.13 for oscillation ones [60]. This similarity comes from the fact that the coverage of the sample was not uniform, the adhesion and incorporation of Chromia–Silica composite powder, as it could be observed in the SEM images being very low, had a very high percentage both in the P6–6420–9 passes test and in the P9–136–9 passes test. This lack of deposition is due to the thermal behavior of chromium oxide, which is found in a very high percentage both in the P6–6420–9 passes and P9–136–9 passes samples.

Table 5 shows the results of scratch testing in the case of the three samples covered with ceramic micropowders.

### 3.5. Microindentation Test

**Figure 10:**
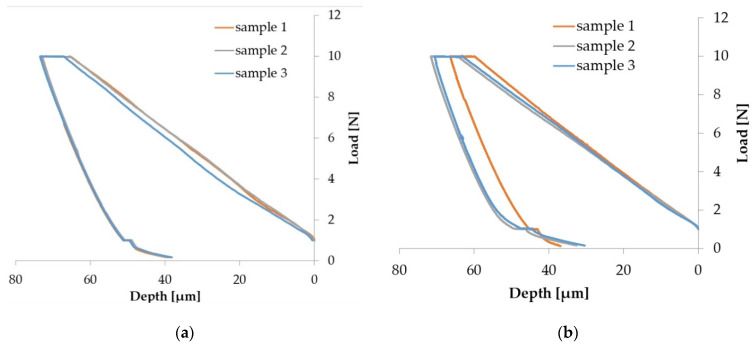

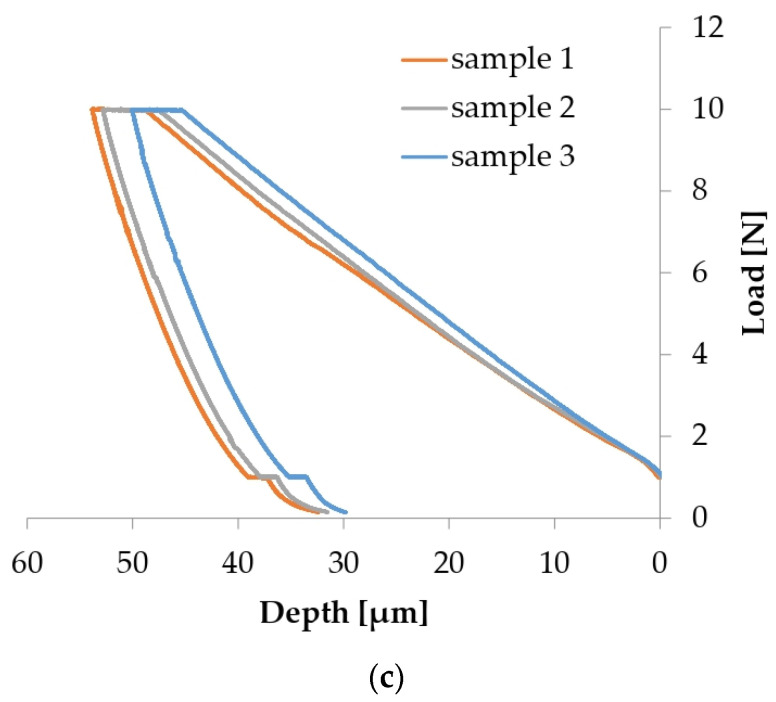
Results of microindentation tests for samples coated with ceramic micropowders: (**a**) P3–143–9 passes; (**b**) P6–6420–9 passes; (**c**) P9–136–9 passes.

To conduct the microindentation test, three samples were tested for each type of ceramic powder used to coat the Arboblend V2 Nature biopolymeric material. Repeated testing was to confirm the experimental stability. Figure 10 shows the evolution of the force as a function of the depth of penetration for all three samples subjected to analysis. The software package used (UMT Test Viewer, 2.16) allowed the reading of both the microhardness values and the Young’s modulus. These values are presented in Table 6.

The force applied to the indenter increases constantly during the charging phase and is maintained at the maximum value of 10 N. This phase is called creep, after which there is a decrease to zero in the discharge phase.

According to the data obtained from the micro-indentation, the P9–136–9 passes sample, although it does not have a uniform coating, presents the best values of microdurity, (0.17 ± 0.01 GPa), the maximum indentation depth being 52.42 ± 1.77 µm. Compared to the zirconium-based ceramic coating (P3–143–9 passes), the chromium oxide coatings have a higher hardness (P6–6420–9 passes, P9–136–9 passes) [35]. Another justification of the results comes from the reduced layer thickness due to the small dimension of microparticles (P3–143–9 passes) than in the case of the other two types of ceramic micropowders.

The lowest dispersion of the results was obtained in the case of the P3–143–9 passes sample, most likely due to the fact that the deposited ceramic layer was uniform. In addition, the other samples tested did not show large differences.

## 4. Conclusions

The coating of bio-based polymers has gained the attention of researchers worldwide. The purpose of coatings with various layers, be they ceramic, metal, etc., as well as in the case of reinforcements, is to increase the characteristics of the base material so that it responds better to certain industrial applications and can even replace a certain material, such as metal. The coatings have been realized in order to increase the mechanical, tribological and thermal characteristics of the samples (wear, hardness and increase in thermal resistance), thus becoming suitable in applications that require harsh working conditions, especially in the automotive industry. The coating of sample with ceramic microlayers led to the following results:

- A slight increase in the melting point, from 172 °C (base material [35,61]) up to 174 °C, varying depending on the thickness of the deposited layer. The thickness value is closely influenced by the microparticles size that constitute the ceramic powder. Thus, the larger the size of the microparticles, the higher the thermal resistance of the coated material is.
- No significant changes were visible both in terms of temperature range and the amount of mass lost during the thermal degradation, and the differences were also attributed in this situation to the size of the ceramic microparticles.
- Regarding the coatings’ uniformity, the SEM surface analysis indicated a good and uniform incorporation of microparticles in the case of composite powder based on zirconium oxide. The other two ceramic micropowders in contact with the polymer matrix did not reveal a good adhesion due to the lower working temperature than the melting point (2435 °C).
- XRD and EDX analyses highlighted the presence of microceramic layers. Their crystalline/semi-crystalline structure confers hardness; thus, they are suitable for applications that require this feature. The coating with Cr_2_O_3_-xSiO_2_-yTiO_2_ (sample 9) formed the hardest layer (0.17 ± 0.01 GPa), which was demonstrated during the microindentation test. Analyzing the obtained results from the SEM and scratch analyses point of view, it can be concluded that the deposition was not uniform due to the fact that the adhesion between the microparticles of chromium oxide, silicon oxide and titanium oxide is lower than in the case of the other two samples, the average value of the apparent friction coefficient (A-COF) being 0.18 ± 0.08.

According to the obtained results regarding the adhesion of the ceramic layers on the polymer surface, it can be stated that the samples showed strong chemical bonds at the interface between the thin layers and Arboblend V2 Nature bio-based polymer. Thus, these coated materials can be used in industrial applications that require high surface hardness and thermal resistance. They can also successfully replace various non-biodegradable polymeric materials used in various applications such as those in the automotive and electronics industry (telephone covers, housings, worm wheels, car wiper system, etc.).

## Figures and Tables

**Table 1:** Technological parameters used during the counting process.

Powder	Gun Type	N_2_		H_2_		Electric		9MP Powder Dispenser			Spray Distance (mm)
		Pressure (Bar)	Gas Flow (NLPM)	Pressure (Bar)	Gas Flow (NLPM)	DC (A)	DC (V)	Carrier Gas Flow (NLPM)	Air Pressure (Bar)	Amount (g/min)	
ZrO_2_ 18TiO_2_ 10Y_2_O_3_	9MB	3.4	44	3.4	6.6	400	70–80	5.3	1.4	144	137
Cr_2_O_3_		3.6	39	3.6	6.6	400	70–80	5.1	1.4	126	145
Cr_2_O_3_-xSiO_2_-yTiO_2_		3.7	42	3.7	6.6	400	70–80	5.1	1.4	132	145

**Table 2:** Experimental plan used to cover the samples with ceramic layer.

No.crt.	Sample Number	Powder Type	Number of Passes
1	1	143	5
2	2	143	7
3	3	143	9
4	4	6420	5
5	5	6420	7
6	6	6420	9
7	7	136	5
8	8	136	7
9	9	136	9

**Table 3:** Calorimetric characterization of samples coated with ceramic layers.

Sample	Transformation	T_onset_ [°C]	T_peak_ [°C]	T_end_ [°C]	ΔH/m [kJ/kg]
P3–143–9 passes	I^st^	62.0	64.7	68.5	−8.81
	II^nd^	81.9	87.2	93.0	19.36
	III^rd^	162.7	169.3	175.3	−40.1
P6–6420–9 passes	I^st^	62.1	65.3	71.2	−4.29
	II^nd^	81.4	87.2	94.2	7.16
	III^rd^	164.0	173.7	185.3	−38.14
P9–136–9 passes	I^st^	61.8	65.8	71.8	−5.57
	II^nd^	81.7	88.7	95.6	8.66
	III^rd^	166.0	171.4	178.0	−48.38

**Table 4:** Thermogravimetric characteristics of the samples covered with ceramic micropowders.

Sample	Stage	T_onset_ [°C]	T_peak_ [°C]	T_end_ [°C]	W [%]	DTA Characteristic	Residue [%]
P3–143–9 passes	I	289	341	369	84.98	exo	3.81
	II	413	423	436	11.21	exo	
P6–6420–9 passes	I	282	346	373	84.61	exo	6.60
	II	413	426	438	8.79	exo	
P9–136–9 passes	I	281	347	367	88.06	exo	1.68
	II	415	426	438	10.26	exo	

T_onset_, the temperature at which thermal degradation begins at each stage; T_end_, the temperature at which the thermal degradation ends at each stage; T_peak_, the temperature at which the degradation rate at each stage is maximum; W%, percentage mass loss at each stage; residue, the amount of degraded sample remaining at a temperature above 700 °C.

**Table 5:** Values of A-COF recorded by samples covered with ceramic layers.

Sample	A-COF Medium Value	A-COF Maximum	Time of A-COF Maximum[s]
P3–143–9 passes	0.29 ± 0.16	0.53	50
P6–6420–9 passes	0.56 ± 0.42	1.62/1.37	3.0/60
P9–136–9 passes	0.18 ± 0.08	0.37	60

**Table 6:** Results obtained by microindenting the samples coated with ceramic micropowders.

Sample	Test	Max Load (N)	Max Depth (µm)	Young’s Modulus (GPa)	Micro Hardness (GPa)
P3–143–9 passes	1	8.99	73.55	1.52	0.11
	2	8.7	72.78	1.69	0.11
	3	8.98	73.02	1.48	0.11
Average		8.98 ± 0.01	73.12 ± 0.4	1.56 ± 0.11	0.11 ± 0.00
P6–6420–9 passes	1	8.99	66.64	1.69	0.13
	2	8.98	71.70	1.91	0.11
	3	8.98	71.02	2.53	0.10
Average		8.99 ± 0.01	69.79 ± 2.75	2.04 ± 0.44	0.12 ± 0.01
P9–136–9 passes	1	8.99	53.77	2.92	0.16
	2	8.99	53.08	3	0.16
	3	8.97	50.42	2.9	0.17
Average		8.99 ± 0.01	52.42 ± 1.77	2.94 ± 0.05	0.17 ± 0.01

## Data Availability

The data presented in this study are available on request from the corresponding author.

## Nomenclature

TPS	Titanium Plasma Spraying
APS	Atmospheric Plasma Spray
PDMS	Polydimethylsiloxane
PDC	biodegradable multiblock copolymer
TFX	Polyetherurethane
PLA	polylactic acid
NLPM	normal litre per minute
DC	Direct Current
DSC	Differential scanning calorimetry
RT	room temperature
TG	Thermogravimetric curves
DTG	derived thermogravimetric curves
DTA	differential thermal analyzes
T_onset_	starting temperature
T_peak_	middle temperature
T_end_	finish temperature
SEM	Scanning Electron Microscopy
LFD	Large Field Detector
EDX	Energy-dispersive X-ray spectroscopy
XRD	X-ray diffraction analysis
W%	percentage mass loss at each stage
A-COF	apparent friction coefficient
Y	testing distance (mm)
